# When Educational Material Is Delivered: A Mixed Methods Content Validation Study of the Information Assessment Method

**DOI:** 10.2196/mededu.6415

**Published:** 2017-03-14

**Authors:** Hani Badran, Pierre Pluye, Roland Grad

**Affiliations:** ^1^ Information Technology Primary Care Research Group Department of Family Medicine McGill University Montreal, QC Canada; ^2^ Herzl Family Practice Centre Department of Family Medicine McGill University Montreal, QC Canada

**Keywords:** validity and reliability, continuing education, Internet, electronic mail, physicians, family, knowledge translation, primary health care

## Abstract

**Background:**

The Information Assessment Method (IAM) allows clinicians to report the cognitive impact, clinical relevance, intention to use, and expected patient health benefits associated with clinical information received by email. More than 15,000 Canadian physicians and pharmacists use the IAM in continuing education programs. In addition, information providers can use IAM ratings and feedback comments from clinicians to improve their products.

**Objective:**

Our general objective was to validate the IAM questionnaire for the delivery of educational material (ecological and logical content validity). Our specific objectives were to measure the relevance and evaluate the representativeness of IAM items for assessing information received by email.

**Methods:**

A 3-part mixed methods study was conducted (convergent design). In part 1 (quantitative longitudinal study), the relevance of IAM items was measured. Participants were 5596 physician members of the Canadian Medical Association who used the IAM. A total of 234,196 ratings were collected in 2012. The relevance of IAM items with respect to their main construct was calculated using descriptive statistics (relevance ratio R). In part 2 (qualitative descriptive study), the representativeness of IAM items was evaluated. A total of 15 family physicians completed semistructured face-to-face interviews. For each construct, we evaluated the representativeness of IAM items using a deductive-inductive thematic qualitative data analysis. In part 3 (mixing quantitative and qualitative parts), results from quantitative and qualitative analyses were reviewed, juxtaposed in a table, discussed with experts, and integrated. Thus, our final results are derived from the views of users (ecological content validation) and experts (logical content validation).

**Results:**

Of the 23 IAM items, 21 were validated for content, while 2 were removed. In part 1 (quantitative results), 21 items were deemed relevant, while 2 items were deemed not relevant (R=4.86% [N=234,196] and R=3.04% [n=45,394], respectively). In part 2 (qualitative results), 22 items were deemed representative, while 1 item was not representative. In part 3 (mixing quantitative and qualitative results), the content validity of 21 items was confirmed, and the 2 nonrelevant items were excluded. A fully validated version was generated (IAM-v2014).

**Conclusions:**

This study produced a content validated IAM questionnaire that is used by clinicians and information providers to assess the clinical information delivered in continuing education programs.

## Introduction

### Theoretical Model and Development of the Information Assessment Method

This paper reports the content validation of an original method for assessing the value of educational material delivered to the health professionals from their perspective. Numerous clinically relevant research studies are published daily; thus, it is impossible for health professionals to filter and absorb all this information. Educational programs strive to overcome this issue, through Web-based information resources and email alert services. In particular, clinical emailing channels deliver educational material to health professionals, such as a Daily POEM research synopsis (POEM stands for Patient-Oriented Evidence that Matters) or a Highlight (a weekly email with evidence-based treatment recommendation) [1-3]. As shown in an earlier article, family physicians perceive advantages from receiving educational material via email [4].

**Figure 1:**
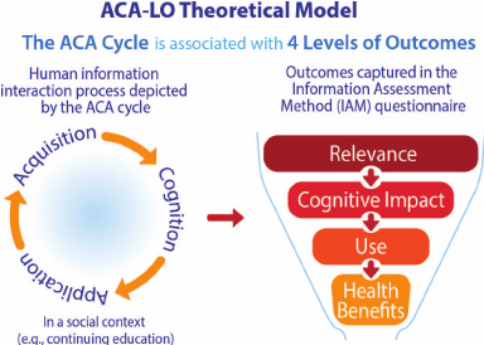
The Acquisition Cognition Application – Levels of Outcome (ACA-LO) theoretical model (reproduced by the permission of the American Board of Family Medicine) [3].

The purpose of this study was to validate a method for assessing the perceived value of information (educational material) delivered by email from the perspective of family physicians (information users). The Information Assessment Method (IAM) is used by more than 15,000 Canadian pharmacists and physicians as a continuing education tool for assessing (reflective learning) outcomes of information delivered in educational programs. The physicians described in this study participate in the longitudinal Daily POEMs program, sponsored by the Canadian Medical Association. This program is certified for continuing medical education credit by the College of Family Physicians of Canada and the Royal College of Physicians and Surgeons of Canada. For each completed IAM questionnaire (reflective learning activity), physicians earned credits. Then, we used the IAM ratings for this validation study. Saracevic and Kantor [5] defined the perceived value of information as an “Acquisition-Cognition-Application” process; subsequently, we linked this process to 4 levels of outcome of information in a theoretical model, which has been operationalized by the IAM questionnaire. Presented elsewhere, the ACA-LO (Acquisition Cognition Application – Levels of Outcome) model explains the “value” of information, that is, how information is valuable from the users’ viewpoint [6-8]. Health professionals subscribe to an alerting service and then acquire a passage of text (acquisition), which they read, understand, and integrate (cognition). Subsequently, they may use this newly understood and cognitively processed information for a specific patient (application). The corresponding subsequent 4 levels of outcomes are as follows: the situational relevance of the information (level 1), its cognitive impact (level 2), the use of this information (level 3), and subsequent health benefits (level 4; Figure 1).

The IAM is a systematic and comprehensive method to assess information from the perspective of the information users; different versions of the IAM questionnaire have been developed for and used by the public (patients and parents) and health professionals (nurses, pharmacists, and physicians) [1,2,7-13]. The IAM can help assess electronic knowledge resources in the context of the “pull” or the “push” of information. A “push-pull acquisition-cognition-application” of information conceptual framework has been published elsewhere [2,14]. On the one hand, “pull” refers to information-seeking behavior, such as a search for information in an electronic knowledge resource. “Push,” on the other hand, refers to information delivery and is currently used in multiple health domains such as continuing education, disease prevention, health education, medical treatment, and nutrition [1,10,15-19]. This is a type of passive acquisition of information such as email alerts.

With respect to the physicians’ evaluation of clinical information in a “push” context, the 2011 version of the IAM questionnaire (IAM-v2011) contained 23 items distributed on 4 constructs (derived from the 4 levels of outcomes): (1) the “cognitive impact” construct contains 6 items of positive impact and 4 items of negative impact (cognitive impact of information on clinicians), (2) the “clinical relevance” construct contains 3 items (relevance of information for a specific patient), (3) the “clinical use” construct contains 7 items (information use for a specific patient), and (4) the “health benefits” construct contains 3 items (expected health benefits for a specific patient; Multimedia Appendix 1). In a “push” context, clinical information will in some way impact a clinician’s continuing education in general (eg, learning something new about a medical intervention) but may not be necessarily relevant for a clinician’s specific patient (in contrast to the “pull” context where clinicians typically seek information for a situation linked to the care of a specific patient). Thus, we sequenced the IAM questions in a pragmatic order (rather than a theoretical order); as such, questions that operationalize the “cognitive impact” construct (level 2) were presented before questions regarding the “clinical relevance” construct (level 1). Hereafter, we follow this pragmatic order. Specifically, the IAM questionnaire has been refined iteratively since 2001 through literature reviews, qualitative, quantitative, and mixed methods research [20]. It allows information users, including professionals, to systematically report these outcomes for each piece of information such as one educational email. For example, in the context of lifelong learning, 13,444 family physician members of the College of Family Physicians of Canada used the IAM to stimulate reflective learning and earn continuing education credits between January 2010 and December 2014 [1]. This process allowed them to rate Highlights that are weekly treatment recommendations from a reference Web-based resource called RxTx. Along with ratings, participants provided constructive feedback to the information provider (the Canadian Pharmacists Association), which was then used to improve the information content of RxTx [21]. This paper addresses the following problem: the IAM has not been fully validated in the “push” context (for information delivery). Regarding the IAM-v2011 for the “push” context, items were developed in line with guidance from Haynes et al [22]. In previous work, we conducted discussions with experts, as well as literature reviews, qualitative, quantitative, and mixed methods research studies [1,2,9,11,21,23-27]. In this paper, we report an evaluation of the content validity of the IAM-v2011.

### Literature Review

One important aspect of the content validation of an assessment tool such as the IAM questionnaire is to ensure that all aspects of the measure are covered [22]. Hence, we reviewed the literature (qualitative, quantitative, and mixed methods studies) about outcomes associated with educational email alerts. The included studies were (1) primary research studies, (2) on educational emails directed to physicians, (3) on outcomes of emails, and (4) reported in English. Specifically, we included the 5 research studies that were included in a 2010 review [2] and tracked research papers (up to March 2014) cited by or citing these studies and 3 literature reviews on educational emails (using the Scopus comprehensive bibliographic database). In addition, we conducted personal searches, for example, in Google Scholar. In total, 258 records were identified (146 from Scopus and 112 from personal searches). Full-text publications were retrieved and screened. A total of 13 studies were included [11,14,26-36]. The included studies had diverse designs: 6 quantitative descriptive studies, 2 randomized controlled trials, 2 qualitative research studies, 2 mixed methods research studies, and 1 quantitative prospective observational study. A thematic synthesis was conducted, and the findings are presented in Table 1. Regarding the outcomes of information constructs, (1) “cognitive impact” was reported in 9 studies, (2) “clinical relevance” was reported in 6 studies, (3) “clinical use” was reported in 8 studies, and (4) “health benefits” was reported in 5 studies. No other construct was reported. No instrument similar to the IAM was found in the literature. Our synthesis supported the 4 constructs covered in the IAM questionnaire, when educational emails are delivered to physicians. Therefore, this paper is aimed to evaluate the content validity of the IAM-v2011 from the perspective of physicians who use the IAM in the context of educational material delivered to physicians.

**Table 1:** Description of the included studies.

Author (year), study title, country	Study design, setting, participants, data collection, data analysis	Intervention	Relevant outcomes	Reported level of outcome
Cook et al (2013), Features of Effective Medical Knowledge Resources to Support Point of Care Learning: A Focus Group Study, Australia [32]	Design: qualitative study. Intervention and setting: 11 focus groups at an academic medical center. Participants: 50 primary care and subspecialist internal medicine and family physicians. Data analysis: comparative inductive thematic	Focus group interview	Features that influence users' selection of knowledge resources: (1) comprehensiveness, (2) search ability and brevity, (3) integration with clinical workflow, (4) credibility, (5) user familiarity, (6) capacity to identify a human expert, (7) reflection of local care processes, (8) optimization for the clinical question (eg, diagnosis, treatment options, drug side effect), and currency, and (9) ability to support patient education	Cognitive impact, information use, clinical relevance, health benefits
Ebell and Grad (2012), Top 20 Research Studies of 2011 for Primary Care Physicians, United States and Canada [31]	Design: a longitudinal Web-based summary of the most relevant, practice-changing POEMs^a^ from 2011 as determined by Canadian raters using IAM-v2011.	Review	Based on IAM^b^ user ratings, these 20 POEMs contain information that is most relevant for primary care physicians	Clinical relevance
Ebell and Grad (2013), Top 20 Research Studies of 2012 for Primary Care Physicians, United States and Canada [33]	Design: a longitudinal Web-based summary of the most relevant, practice-changing POEMs from 2012 as determined by Canadian raters using IAM-v2011.	Review	Based on IAM user ratings, these 20 POEMs contain information that has cognitive impact, is clinically relevant, is used, has health benefits for the patient, and is most relevant for primary care physicians	Clinical relevance
Galvao et al (2013), The Clinical Relevance of Information Index (CRII): Assessing the Relevance of Health Information to the Clinical Practice, Canada [27]	Design: a longitudinal Web-based study. Data collection: IAM rating of physicians in response to educational emails. CRII was applied to 4574 relevance assessments of 194 evidence synopses sent by email. Participants: 41 family physicians in 2008. Data analysis: descriptive statistical analysis.	Educational emails	The CRII is only weakly correlated with the number of citations received by a study and the level of evidence of the study. The CRII captures aspects of information not considered by other indices to be used by information providers, institutions, editors, as well as health and information professionals targeting knowledge translation.	Clinical relevance
Grad et al (2011), Do Family Physicians Retrieve Synopses of Clinical Research Previously Read as Email Alerts? Canada [14]	Design: mixed methods study. Participants: 41 family physicians. Settings: 9 different provinces of Canada. Intervention: IAM linked to POEM emails and searches in Essential Evidence Plus. Data collection: QUAN^c^: Pull, from PDA, Push, from IAM of POEMs. QUAL^d^: interview. Analysis: QUAN: descriptive statistics, QUAL: thematic.	Educational emails and face-to-face interviews	Family physicians purposefully retrieved a synopsis they had previously read as email. Factual knowledge from brief reading of email alerts of synopses may be simply forgotten. The ability of family physicians to remember synopses they previously read declined over time.	Cognitive impact
Law et al (2008), Facilitating Knowledge Transfer Through the McMaster PLUS REHAB Project: Linking Rehabilitation Practitioners to New and Relevant Research Findings, Canada [30]	Design: a longitudinal Web-based study. Setting: Mac-PLUS REHAB project, Canada. Participants: 1000 practicing occupational therapists and physiotherapists. Data collection: email alerts about new evidence tailored to the users’ interest profile allow them to interact and submit feedback. Data analysis: descriptive statistical analysis.	Educational emails	PLUS REHAB: (1) helps occupational health professionals access and uptake of information, (2) speeds up the knowledge transfer process, (3) supports practice and knowledge sharing, (4) evaluates the effect of push-out technology on uptake and use of evidence-based knowledge, and (5) makes knowledge accessible by individualizing alerts, providing a credibly rated and trustworthy system of relevant articles and saving many valuable hours.	Cognitive impact, information use, clinical relevance
Leung et al (2010), A Reflective Learning Framework to Evaluate CME Effects on Practice Reflection, Canada [34]	Design: qualitative multiple case study. Participants: 473 practicing family physicians commented on research-based synopses after reading and rating them as an online (pull and push) CME learning activity. Data collection: these comments formed 2029 cases from which cognitive tasks were extracted. Data analysis: thematic analyses and cross-case analysis.	Internet (push) educational activities	Four cognitive processes and 12 cognitive tasks were supported. Reflective learning was defined as 4 interrelated cognitive processes: (1) interpretation, (2) validation, (3) generalization, and (4) change. Reflective learning performances of family physicians were evaluated.	Cognitive impact
McMullin and Singh (2006), A Single Email to Clinicians May Improve Short-Term Prescribing for People With Coronary Artery Disease and Raised LDL^e^ Cholesterol, United States [29]	Design: randomized trial. Participants and settings: 14 US primary care physicians in academically affiliated practice. Data collection: physicians were blinded to group allocation. Intervention and data collection: intervention group received a single email, provided decision support, and facilitated “one-click” actions such as prescriptions, updating charts, and mailing out educational materials. Data analysis: descriptive statistical analysis.	Educational emails	The intervention group participants were more likely than controls to change their prescription. Median time to the first medication adjustment was earlier in the intervention group. LDL cholesterol levels for people with baseline levels greater than 130 mg/dL were significantly lower in the intervention group (119 vs 138.0 mg/dL). It took physicians less than 1 minute to process each email. A single email to primary care physicians could influence prescribing and may improve hyperlipidemia management in the short term.	Information use, health outcome
Pluye et al (2010), Evaluation of Email Alerts in Practice: Part 2 – Validation of the Information Assessment Method, Canada [26]	Design: mixed methods sequential explanatory. Data collection: a daily educational email was sent to 12,800 doctors. Participants: 1007 family doctors who submitted 61,493 ratings of “cognitive impact” (QUAN) and 46 doctors were interviewed (QUAL). Setting: Canada (QUAN), McGill academic setting (QUAL). Data analysis: descriptive statistical analysis (QUAN) and deductive thematic analysis (QUAL).	Educational emails and face-to-face interview	IAM contributes to: (1) research for systematically assessing and comparing the relevance, cognitive impact, use, and expected health outcomes associated with email alerts; (2) continuing professional development for documenting brief individual e-learning activities; and (3) two-way knowledge exchange between information providers and clinicians for improving email alerts.	Cognitive impact, clinical relevance, information use, health benefits
Pluye et al (2012), Feasibility of a Knowledge Translation CME Program: Courriels Cochrane, Canada [11]	Design: a longitudinal evaluation study. Data collection: participants received weekly emails with synopses of Cochrane reviews and rated them using the IAM. Participants: 985 French-speaking family physicians. Setting: Canada. Data analysis: statistical descriptive analysis	Educational emails and IAM questionnaire	Of 1109 completed questionnaires: 87.7% reported positive cognitive impact.75.3% reported the information was clinically relevant.53.7% reported that information use.51.3% of ratings contained reports of information use was associated with health benefits	Cognitive impact, clinical relevance, information use, health benefits
Schopf and Flytkjær (2012), Impact of Interactive Web-Based Education With Mobile and Email-Based Support of General Practitioners on Treatment and Referral Patterns of Patients with Atopic Dermatitis: Randomized Controlled Trial, Norway [36]	Design: randomized controlled trial. Participants: general practitioners, Norway. Intervention: a Web-based course on atopic dermatitis with guidance via email from specialists. Data collection: 46 physicians: 24 doctors were allocated to the intervention group and 22 doctors to the control group. Data analysis: descriptive statistical analysis.	Educational emails	There were no statistically significant differences in the duration of topical steroid treatment or number of treatment modalities between the groups. The lack of effect on the primary outcome may be due to attrition as 54% of the participants did not complete the course; 42% (10/24) of physicians sent at least one educational request via email. While 11% (8/73) of treatment reports in the intervention group were referred to a medical specialist (eg, dermatologist or pediatrician).	Information use, health benefit
Strayer et al (2010), Updating Clinical Knowledge: An Evaluation of Current Information Alerting Services, United States [28]	Design: Web-based study. Data collection: a 7-item checklist (push tools) based on evidence-based medicine was created and assessed for content validity and face validity. Participants: practicing clinicians, clinician researchers, and experts (n=7). Data analysis: descriptive statistics analysis	Educational emails information assessment tool	A checklist was created and can be used to reliably assess the quality of clinical information updating (push) tools. This tool will improve the application of basic evidence-based medicine principles to new medical information in order to increase their usefulness to clinicians.	Cognitive impact, information use
Wang et al (2009), The Cognitive Impact of Research Synopses on Physicians: A Prospective Observational Analysis of Evidence-Based Summaries Sent by Email, Canada [35]	Design: prospective observational study. Intervention and data collection: research synopses sent by email. Each synopsis was classified as either positive or negative based on physician-reported impacts. A total of 1960 Canadian physicians submitted 159,442 ratings on 193 synopses. Each synopsis was assessed on average by 826 physicians. Participants and setting: physicians, Canada. Data analysis: statistical analysis descriptive and logistic regression.		There were 28.3 negative ratings per research synopsis, 146.3 neutral, and 656.2 positive. Out of the 7 characteristics (number of characters, research design, study setting, number of types of patient populations studied, number of comparisons, number of outcomes, and number of results) analyzed, only the number of comparisons had a statistically significant influence on physician ratings. An increase in the number of comparisons or the number of results decreased the likelihood of a negative impact. Characteristics of the synopses appear to influence cognitive impact, and there might be lexical patterns specific to these factors.	Cognitive impact

^a^POEM: Patient-Oriented Evidence that Matters.

^b^IAM: Information Assessment Method.

^c^QUAN: quantitative.

^d^QUAL: qualitative.

^e^LDL: low-density lipoprotein.

## Methods

### Mixed Methods Design

We used a 3-part mixed methods convergent design (quantitative, qualitative, and mixing) [37,38]. In the quantitative part, the relevance of IAM-v2011 items was measured using data collected from a Web-based longitudinal study. In the qualitative part, we evaluated the representativeness of IAM-v2011 items and their relationship to the IAM constructs. Considering that ecological content validation is determined by the end users [39,40], the viewpoint of actual IAM users was needed, and participants were IAM users in the quantitative and qualitative parts of the validation study. In the mixing part, quantitative and qualitative results were integrated and discussed with experts.

We conducted an evaluation of the ecological and logical content validity of the IAM-v2011. Validity refers to whether a test measures what it is supposed to measure [41-44], and content validity is defined as “the degree to which elements of an assessment instrument are *relevant* to and *representative* of the targeted construct for a particular assessment purpose” [22]. The *relevance* of an assessment instrument refers to the appropriateness of its elements for the targeted construct and function of assessment. For example, the relevance of an item refers to the degree to which this item is likely to accomplish the goal implied by the construct. Relevance can be evaluated through quantitative methods. The *representativeness* of an assessment instrument refers to whether its elements cover all facets of the targeted constructs. For example, a representative item gives a good indication of what its construct is intended to measure. Representativeness can be evaluated through qualitative methods.

Content validity can be divided into (1) logical content validity in which a determination is left to experts and (2) ecological content validity in which the determination is obtained from the users [39]. Ecological validity is the degree to which the behaviors observed and recorded in a study reflect the behaviors that actually occur in natural settings [39]. Our general objective was to assess the logical and ecological content validity of IAM-v2011 for educational email alerts. In line with standard procedures for content validation of evaluation tools [22], our specific objectives were to measure the relevance and evaluate the representativeness of IAM-v2011 items for assessing information received via email alerts.

### Part 1: Quantitative Longitudinal Study

**Figure 2:**
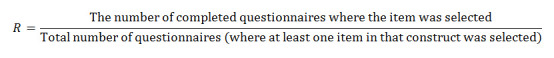
Formula of the relevance ratio (R).

A Web-based longitudinal study was conducted. We considered all 2012 IAM ratings submitted by physicians after reading a Daily POEM email alert [33]. Tailored to a primary care audience, Daily POEMs are synopses of original primary research or systematic reviews, selected after scanning and critically appraising studies published in 102 medical journals. A total of 270 Daily POEMs were emailed to physician members of the Canadian Medical Association in 2012. Participants were all physicians across Canada who subscribed voluntarily to receive Daily POEMs and rated at least one POEM in 2012 using the IAM-v2011 as a requirement to obtain continuing education credit. From 5596 physicians, we collected 234,196 IAM completed Web-based questionnaires (ratings) from January 1 to December 31, 2012. Regarding the data analysis, for each IAM-v2011 item of the construct, a ratio (R) was calculated using the formula shown in Figure 2.

Stated otherwise, for each construct or subconstruct, the relevance ratios of all items were calculated. For example, with regard to the item “I learned something new,” the relevance ratio R was calculated as follows. The number of completed questionnaires where this item was selected was divided by the total number of IAM questionnaires in which at least one item of the “Positive cognitive impact” construct was selected. In line with the standards for educational and psychological testing [45], validation is a joint responsibility of the developer and the knowledge user. IAM knowledge users (users of the results of the analysis of IAM ratings) are information providers (such as the Canadian Pharmacists Association, which produces the abovementioned Highlights) and appreciate the “Negative cognitive impact” items, which can detect issues with information content. Thus, negative cognitive impact items are rarely selected, but necessary, and the construct “cognitive impact” has been divided into 2 subconstructs: “positive” and “negative” cognitive impact. For example, with respect to the item “This information can be harmful,” the number of completed questionnaires where this item was selected was divided by the total number of questionnaires in which at least one item of the construct “Negative cognitive impact” was selected in order to calculate the value of R.

The results were interpreted as follows. In line with our prior content validation study in a “pull” context [46], the items were deemed relevant when R was 10% and above and irrelevant when R was less than 10%. With respect to the cutoff value of R to exclude items, there is no agreed upon criterion or universal cutoff to determine content validity [41,42].

### Part 2: Qualitative Descriptive Study

A qualitative descriptive study was conducted [47] through semistructured face-to-face interviews with 15 family physicians (end users). The interviews started with general questions about educational email alerts and continuing medical education activities, to explore participants’ experiences; then, we asked specific questions on the representativeness of IAM-v2011 items.

#### Participants and Setting

An email invitation was sent to all physician members of the Department of Family Medicine at McGill University (n=269). Our eligibility criteria were (1) practicing family physician working in the greater Montréal area, (2) receiving educational email alerts, and (3) rating Daily POEMs or Highlights using the IAM. Of the 17 family physicians who volunteered, 15 were interviewed, while 2 were excluded (1 had no experience with the IAM-v2011 and 1 was not available).

#### Data Collection

Before each interview, participants received a brief lay summary of the study. For each IAM-v2011 item, participants were asked about its representativeness as follows: (1) the interviewer started by explaining each construct and the definition of that construct, (2) each participant was then asked to read the construct and its corresponding items on paper, and (3) for each construct, the participant was asked open-ended questions about the items and if they were suitable for that construct. For example, the interviewees were asked whether they would add, modify, or delete some items and the reasons behind their opinion. Although focus groups can be used in content validation studies [40], we decided to conduct individual interviews because we were interested mainly in individual experience and perception of the use of the IAM linked to educational emails. Interviews were recorded, reviewed, and transcribed on the same day of the interview. Our interview guide is available on request.

#### Data Analysis

We conducted hybrid deductive-inductive thematic analysis. This type of analysis consists of applying themes (theory-driven) and searching for themes that emerge because of their importance to the description of the phenomenon under study [48]. The inductive process involves the identification of emerging or new themes through “careful reading and re-reading of the data” [49]. We summarized and analyzed the interview transcripts. We assigned preliminary themes based on our ACA-LO theoretical model and the interview guide and then searched for themes that emerged. The coding process was conducted in 6 stages [50,51]: (1) developing a code manual, (2) testing the reliability of codes, (3) summarizing the data and identifying initial themes, (4) applying a template of codes for the meaningful themes, (5) connecting the codes in accordance with the process of discovering patterns in the data, and (6) corroborating and legitimating coded themes. The final results were discussed with 7 members of the Information Technology Primary Care Research Group (ITPCRG) who are experts in the IAM. For each construct, a table was created that contained themes collected from interviews. For each IAM item, we had 8 possibilities. There were 4 initial possibilities (4 deductive themes): (1) addition, (2) deletion, (3) modification of an item, and (4) no change. Then, 4 additional possibilities emerged (4 inductive themes): (1) merge two or more items, (2) merge two or more items and add a new element, (3) keep the main item and delete subitems, and (4) keep the main item and add a new subitem. An item was deemed representative of the corresponding construct when it was confirmed (modified or unchanged) or added (new item). An item was deemed not representative when participants suggested its deletion.

### Part 3: Mixing Quantitative and Qualitative Parts

Qualitative and quantitative results were integrated and compared. Such a comparison of results has been recommended in reference books on mixed methods, specifically in primary care research [37,52]. The relevance and representativeness of IAM items were tabulated. Items of questionable relevance or representativeness were identified and discussed with ITPCRG members. IAM items with low relevance or those that were not representative were excluded. In addition, we reviewed and discussed the clarity and language of all items. A final decision regarding each item was achieved by consensus of ITPCRG members. For excluding items, priority was given to the quantitative data received from the 5596 physicians (relevance). The qualitative findings might have suggested new items (representativeness). In our study, qualitative findings supported the removal of 1 nonrelevant item and corroborated quantitative results but did not suggest any new item.

### Ethical Approval

This study was conducted according to the ethical principles stated in the Declaration of Helsinki. Ethical approval was obtained from the McGill University Institutional Review Board. The Institutional Review Board provided ethical approval #A11-E25-05A for collecting and analyzing the quantitative data and #A06-E44-13A for the qualitative data collection and analysis.

## Results

Results are presented according to the 3 parts of the mixed methods design.

### Part 1: Quantitative Results

Of 23 items, 21 had an R value of greater than 10% (N=234,196). All 21 were kept for proposing a 2014 version of the IAM (IAM-v2014; in Table 2, all items except items 1 and 13). The remaining 2 items had an R value of less than 10% (in Table 2, see items 1 and 13). R was 4.86% (N=234,196) for item 1 of the construct “Positive cognitive impact” (“My practice will be changed and improved”) and 3.04% (n=45,394) for item 13 of the construct “Information use” (“I did not know what to do, and I will use this information to manage this patient”). The final decision for items 1 and 13 was to exclude them.

**Table 2:** Relevance of the Information Assessment Method IAM-v2011 items.

Constructs and items^a^			Number of ratings^b^	Relevance ratio (R), %	Decision^c^
**Positive cognitive impact (N=234,196)**					
	1. My practice is (will be) changed and improved.		11,380	4.86	Delete
	2. I learned something new.		135,055	57.67	Keep
	3. I am motivated to learn more.		51,763	22.10	Keep
	4. This information confirmed I did (am doing) the right thing.		39,383	16.82	Keep
	5. I am reassured.		43,835	18.72	Keep
	6. I am reminded for something I already knew.		34,456	14.71	Keep
**Negative cognitive impact (n=6742)**					
	7. I am dissatisfied.		4190	62.15	Keep
	8. There is a problem with the presentation of this information.		1478	21.92	Keep
	9. I disagree with the content of this information.		1289	19.12	Keep
	10. This information is potentially harmful.		766	11.36	Keep
**Information use (n=45,394)**					
	11. As a result of this information I will manage this patient differently.		10,460	23.04	Keep
	12. I had several options for this patient, and I will use this information to justify a choice.		15,944	35.12	Keep
	13. I did not know what to do, and I will use this information to manage this patient.		1378	3.04	Delete
	14. I thought I knew what to do, and I used this information to be more certain about the management of the patient.		6752	14.87	Keep
	15. I used this information to better understand a particular issue related to this patient.		7894	17.39	Keep
	16. I will use this information in discussion with this patient, or with other health professionals about this patient.		18,135	39.95	Keep
	17. I will use this information to persuade this patient, or to persuade other health professionals to make a change for this patient		5607	12.35	Keep
**Expected health benefits (n=38,753)**					
	18. This information will help to improve this patient’s health status, functioning or resilience (ie, ability to adapt to significant life stressors).		12,935	33.38	Keep
	19. This information will help to prevent a disease or worsening of disease for this patient.		13,522	34.89	Keep
	20. This information will help to avoid unnecessary or inappropriate treatment, diagnostic procedures, preventive interventions or a referral, for this patient.		20,474	52.83	Keep
**Clinical relevance (n=234,193)**					
	21. Totally relevant		82,368	35.17	Keep
	22. Partially relevant		85,227	36.39	Keep
	23. Not relevant		66,500	28.40	Keep

^a^n refers to the number of completed questionnaires where at least one item of the same construct was selected.

^b^Number of ratings per item.

^c^Initial decision based on quantitative results.

### Part 2: Qualitative Results

We interviewed 9 male and 6 female family physicians. A total of 9 participants were working in academic health science centers, while 6 were working in community-based private family medicine clinics. The participants’ number of years in practice ranged from 9 to 38 years. A total of 5 participants indicated no particular clinical focus to their practice, while 10 expressed a special interest such as maternity and newborn care (n=3) or care of the elderly (n=3). We interviewed all participants in their offices. The participants were welcoming and cooperative. Of 15 interviewees, 11 gave ample time for the interview, while 4 seemed rushed. For each IAM-v2011 item, all interviewees answered all our questions about its relationship to its construct and whether they would add, modify, or delete it if they had the option to do so. Results of the qualitative part of the study are presented below (construct by construct) and summarized in Table 3.

**Table 3:** Representativeness of the Information Assessment Method IAM-v2011 items.

Constructs and items			Representative	Decision^a^	
**Positive cognitive impact**			
	1. My practice is (will be) changed and improved.		Yes	Keep
	2. I learned something new.		Yes	Keep
	3. I am motivated to learn more.		Yes	Keep
	4. This information confirmed I did (am doing) the right thing.		Yes	Keep
	5. I am reassured.		Yes	Keep
	6. I am reminded of something I already knew.		Yes	Keep
**Negative cognitive impact**				
	7. I am dissatisfied.		Yes	Keep
	8. There is a problem with the presentation of this information.		Yes	Keep
	9. I disagree with the content of this information.		Yes	Keep
	10. This information is potentially harmful.		Yes	Keep
**Information use**				
	11. As a result of this information I will manage this patient differently.		Yes	Keep
	12. I had several options for this patient, and I will use this information to justify a choice.		Yes	Keep
	13. I did not know what to do, and I will use this information to manage this patient.		No	Delete
	14. I thought I knew what to do, and I used this information to be more certain about the management of this patient.		Yes	Keep
	15. I used this information to better understand a particular issue related to this patient.		Yes	Keep
	16. I will use this information in a discussion with this patient, or with other health professionals about this patient.		Yes	Keep
	17. I will use this information to persuade this patient, or to persuade other health professionals to make a change for this patient.		Yes	Keep
**Expected health benefits**				
	18. This information will help to improve this patient’s health status, functioning or resilience (ie, ability to adapt to significant life stressors).		Yes	Keep
	19. This information will help to prevent a disease or worsening of disease for this patient.		Yes	Keep
	20. This information will help to avoid unnecessary or inappropriate treatment, diagnostic procedures, preventative interventions or a referral, for this patient.		Yes	Keep
**Clinical relevance**			
	21. Totally relevant		Yes	Keep
	22. Partially relevant		Yes	Keep
	23. Not relevant		Yes	Keep

^a^Provisory decision based on qualitative results.

#### Construct “Cognitive Impact”

The 10 IAM-v2011 items associated with this construct were representative. For example, about the item “I am motivated to learn more” (item 3), one interviewee said, “I would like to modify this item to be more specific and to be ‘I am motivated to learn more about this topic.’”

#### Construct “Clinical Relevance”

We asked specific questions about this construct, in particular the item “information partially relevant.” Of 15 participants, 9 participants interpreted this item as follows: some information from a Daily POEM or a Highlight covers an aspect of a patient’s condition, or the information does not exactly fit the patient’s condition. A total of 4 participants said this item can be interpreted as either clinically relevant or not relevant. One participant interpreted this item as “information clinically relevant,” while another participant interpreted it as “information clinically not relevant.”

#### Construct “Information Use”

Of the 7 items associated with this construct, 6 were representative, while 1 item was not. By way of illustration, an interviewee said about the latter (item 13 “I did not know what to do, and I will use this information to manage this patient”): “I would like to delete this item as it is redundant.”

#### Construct “Health Benefits”

All 3 items were representative.

### Part 3: Mixing Quantitative and Qualitative Results

Results of quantitative and qualitative analyses were integrated. All IAM-v2011 items, their relevance, representativeness, and a final decision are presented in Table 4. Decision making involved discussions with ITPCRG members, after which 1 item with a low relevance ratio (item 1) and 1 nonrepresentative item with a low relevance ratio (item 13) were excluded from the IAM. With regard to the former item (representative with low relevance ratio), priority was given to the quantitative data (relevance) because it provided feedback from 5596 users. The 21 other items were deemed relevant and representative. There was no item with a high relevance ratio that was nonrepresentative. No new items were suggested from the qualitative data.

**Table 4:** Mixing quantitative and qualitative results.

Constructs and items			Quantitative results: relevance	Qualitative results: representativeness	Final decision
**Positive cognitive impact**				
	1. My practice is (will be) changed and improved.		Delete	Keep	Delete
	2. I learned something new.		Keep	Keep	Keep
	3. I am motivated to learn more.		Keep	Keep	Keep
	4. This information confirmed I did (am doing) the right thing.		Keep	Keep	Keep
	5. I am reassured.		Keep	Keep	Keep
	6. I am reminded for something I already knew.		Keep	Keep	Keep
**Negative cognitive impact**				
	7. I am dissatisfied.		Keep	Keep	Keep
	8. There is a problem with the presentation of this information.		Keep	Keep	Keep
	9. I disagree with the content of this information.		Keep	Keep	Keep
	10. This information is potentially harmful.		Keep	Keep	Keep
**Information use**					
	11. As a result of this information I will manage this patient differently.		Keep	Keep	Keep
	12. I had several options for this patient and I will use this information to justify a choice.		Keep	Keep	Keep
	13. I did not know what to do, and I will use this information to manage this patient.		Delete	Delete	Delete
	14. I thought I knew what to do, and I used this information to be more certain about the management of this patient.		Keep	Keep	Keep
	15. I used this information to better understand a particular issue related to this patient.		Keep	Keep	Keep
	16. I will use this information in a discussion with this patient or with other health professionals about this patient.		Keep	Keep	Keep
	17. I will use this information to persuade this patient, or to persuade other health professionals to make a change for this patient.		Keep	Keep	Keep
**Expected health benefits**					
	18. This information will help to improve this patient’s health status, functioning or resilience (ie, ability to adapt to significant life stressors).		Keep	Keep	Keep
	19. This information will help to prevent a disease or worsening of disease for this patient.		Keep	Keep	Keep
	20. This information will help to avoid unnecessary or inappropriate treatment, diagnostic procedures, preventive interventions or a referral for this patient.		Keep	Keep	Keep
**Clinical relevance**				
	21. Totally relevant		Keep	Keep	Keep
	22. Partially relevant		Keep	Keep	Keep
	23. Not relevant		Keep	Keep	Keep

## Discussion

### Principal Findings

These results have led us to produce a 21-item content validated version of the IAM for “push” technology, presented in Multimedia Appendix 2 (IAM-v2014). This work contributes to advance knowledge in continuing education, and continuing education tools, as there are no similar methods reported in the literature. Outside email alerts, our results can be applied to other Web-based means that deliver educational material, such as apps on mobile devices. For example, we have developed an app (called IAM Medical Guidelines) providing spaced education in a continuing medical education program on respiratory diseases. In such a program, the IAM questionnaire is used by clinicians to document reflective learning and earn continuing education credits.In addition, these results contribute to practice at 3 levels (user, provider, and researcher). First, at the level of the individual knowledge user, physicians can use a validated method to assess the clinical information delivered to them through educational email alerts. More than 15,000 Canadian family physicians and pharmacists are using the validated version of the IAM questionnaire to assess educational email alerts and earn continuing education credits in programs such as Daily POEMs and Highlights. During the calendar year of 2016, the IAM questionnaire (push version) was completed more than 400,000 times by physicians and pharmacists in Canada. To our knowledge, the IAM questionnaire is the most frequently used questionnaire in Canada, in the context of the continuing education of health professionals. Second, at the organizational knowledge provider level, the analysis of IAM-v2014 ratings can be based on a validated method. For example, information providers such as the Canadian Pharmacists Association are receiving validated feedback from their members. Third, using a validated questionnaire offers at least two other advantages: (1) researchers will save time and resources by avoiding the lengthy process of developing and validating their own instrument, and (2) new studies can compare their findings against those of other IAM-based studies.

### Limitations of Our Study

The validation of the IAM as a whole is based on our prior work and a theoretical model, although we gathered quantitative and qualitative evidence for validating each construct and item. Future research may pursue the validation of the IAM as a whole, for example, using factor analysis. As mentioned in the standards for educational and psychological testing, validation can always be pursued [45]. With respect to the quantitative part of the study, as continuing education programs rely on the voluntary participation of physicians, we acknowledge a selection bias with respect to the participants. While our quantitative data sample comprised 234,196 IAM questionnaires completed by 5596 physicians, these participants were not representative of all Canadian physicians. For example, participants were more likely to be comfortable with information technology. With respect to the qualitative part, although focus groups are sufficient for content validation [40], we chose to conduct face-to-face interviews as it is typically difficult to arrange meetings with groups of physicians.

Our data regarding the expected patient health benefits of clinical information reflect the subjective views of health care professionals. For example, a limited number of studies report how using information from knowledge resources may have helped physicians to avoid unnecessary tests, treatments, or referral to specialist colleagues. Outside research conducted in computer laboratories using clinical scenarios, most of the studies share the limitation of self-report and do not objectively examine patient-related outcomes. With respect to the literature on continuing education in the health professions, basing study outcomes on self-report is typical. For instance, a scoping review examined the impact of physician self-audit programs [53]. None of the 6 observational studies included in the review objectively assessed outcomes. To the extent that self-report encourages socially desirable responses, the validity of study outcomes based on self-reported behavior and expected health benefits for patients can be questioned in future research.

### Strengths of Our Study

Our content validation study followed the usual recommendations for developing psychometric and educational assessment tools [22,39]. In previous work, we reviewed information studies and developed a theoretical model, while in this study we gathered quantitative and qualitative evidence to support the use of the IAM in a specific context: the delivery of educational material. Content validation is typically a mixed methods research endeavor [37,38,54]. On the basis of the complementarity and synergy between qualitative and quantitative methods, mixed methods enhance validation studies by integrating quantitative and qualitative results on different aspects of the instruments. For example, focus groups provide qualitative evidence on relevance and representativeness of concepts [40], which are then tested using factor analysis (providing quantitative evidence on convergent and discriminant concepts).

Our validation study was based on Messick’s definition of validity [42-44], which still informs the standards for educational and psychological testing [45]. Our mixed methods study assessed the content validity of the IAM. For each construct, we used quantitative methods to measure the relevance of IAM items and qualitative methods to evaluate their representativeness; then, we integrated the quantitative and qualitative results. In case of divergence, we gave more weight to quantitative results with respect to final decisions about “deleting” an item because the quantitative sample was large. In addition to the large sample in the quantitative part of the study, we interviewed 15 physician users of the IAM. This can be considered as a consultation with ecological experts (IAM users) [40]. The final steps in our data analysis and the draft of IAM-v2014 were discussed with ITPCRG members who are logical experts on assessing the value of clinical information. Expert panel discussion is a core component of content validation [22].

### Conclusions

This study produced a content validated IAM questionnaire (IAM-v2014) that is used by clinicians and information providers to assess the clinical information delivered in continuing education programs. Research on how the quality of health care and the health of specific patients are associated with the delivery of educational content can use tools to accurately document clinical events at multiple points in time. One of the tools for researchers to conduct this type of work is our validated IAM questionnaire, coupled with data from electronic medical records. Finally, the IAM can facilitate a continuous interactional process between information providers who deliver “best” evidence (knowledge translation) and information users who assess this evidence (ratings) and submit constructive feedback; in turn, information providers may use this feedback from information users to optimize their evidence (thereby establishing two-way knowledge translation), which can be made available on the Internet for further retrieval [21]. Using the IAM, the delivery of research-based educational information can be enhanced by experience-based information from health professionals. For example, in addition to the IAM ratings, health professionals provide a substantial amount of free-text comments. These comments include constructive feedback such as suggestions for additional content, reservation or disagreement, suggestions to consider contradictory evidence, or a need for clarification of content. This two-way knowledge translation appears to be unique with regard to information management [55]. In line with the literature on relational marketing [56], being open to user feedback and handling such feedback can improve an educational resource and aid information providers in sustaining relationships with the users by valuing their expertise.

## Appendix

Multimedia Appendix 1 The 2011 version of the Information Assessment Method (IAM-v2011) in a “push” context.

Multimedia Appendix 2 The 2014 version of the Information Assessment Method (IAM-v2014) in a “push” context.

## Abbreviations

ACA-LO: Acquisition Cognition Application – Levels of Outcome
IAM: Information Assessment Method
ITPCRG: Information Technology Primary Care Research Group
POEM: Patient-Oriented Evidence that Matters
