# Supplementary material for: When Educational Material Is Delivered: A Mixed Methods Content Validation Study of the Information Assessment Method
Source: JMIR Med Educ. 2017 Mar 14;3(1):e4. doi: 10.2196/mededu.6415 (PMC5373673; doi:10.2196/mededu.6415)
Supplement: Multimedia Appendix 1 [file mededu_v3i1e4_app1.pdf]

Construct 'Cognitive Impact'
What is the impact of this information on you or your practice?
Positive cognitive impact items:
1) My practice is (will be) changed and improved. If Yes, what aspect is (will be) changed or improved?
2) I learned something new
3) I am motivated to learn more
4) This information confirmed I did (am doing) the right thing
5) I am reassured
6) I am reminded of something I already knew

Negative cognitive impact items:
7) I am dissatisfied
8) There is a problem with the presentation of this information
9) I disagree with the content of this information
10) This information is potentially harmful

Construct 'Clinical Relevance'
Is this information relevant for at least one of your patients?
Items:
11) Totally relevant
12) Partially relevant
13) Not relevant

Construct 'Clinical Use'
Will you use this information for a specific patient?
Items:
14) As a result of this information I will manage this patient differently
15) I had several options for this patient, and I will use this information to justify a choice
16) I did not know what to do, and I will use this information to manage this patient
17) I thought I knew what to do, and I used this information to be more certain about the management of this patient
18) I used this information to better understand a particular issue related to this patient
19) I will use this information in a discussion with this patient, or with other health professionals about this patient
20) I will use this information to persuade this patient, or to persuade other health professionals to make a change for this patient

Construct 'Health Benefits'
For this patient, do you expect any health benefits as a result of applying this information?
Items:
21) This information will help to improve this patient's health status, functioning or resilience (i.e., ability to adapt to significant life stressors)
22) This information will help to prevent a disease or worsening of disease for this patient
23) This information will help to avoid unnecessary or inappropriate treatment, diagnostic procedures, preventative interventions or a referral, for this patient.
