# Supplementary material for: When Educational Material Is Delivered: A Mixed Methods Content Validation Study of the Information Assessment Method
Source: JMIR Med Educ. 2017 Mar 14;3(1):e4. doi: 10.2196/mededu.6415 (PMC5373673; doi:10.2196/mededu.6415)
Supplement: Multimedia Appendix 2 [file mededu_v3i1e4_app2.pdf]

What is the impact of this information on you or your practice?

- ☐ I learned something new.
- ☐ I am motivated to learn more.
- ☐ This information confirmed I did (am doing) the right thing.
- ☐ I am reassured.
- ☐ I am reminded for something I already knew.
- ☐ I am dissatisfied.
- ☐ There is a problem with the presentation of this information.
- ☐ I disagree with the content of this information.
- ☐ This information is potentially harmful.

Is this information relevant for at least one of your patients?

- ☐ Totally relevant
- ☐ Partially relevant
- ☐ Not relevant

Will you use this information for a specific patient?

- ☐ As a result of this information I will manage this patient differently.
- ☐ I had several options for this patient and I will use this information to justify a choice.
- ☐ I thought I knew what to do, and I will use this information to be more certain about the management of the patient.
- ☐ I will use this information to better understand a particular issue related to this patient.
- ☐ I will use this information in a discussion with this patient or with other health professionals about this patient.
- ☐ I will use this information to persuade this patient, or to persuade other health professionals to make a change for this patient

For this patient, do you expect any health benefits as a result of applying this information?

- ☐ This information will help to improve this patient's health status, functioning or resilience (i.e. ability to adapt to significant life stressors)
- ☐ This information will help to prevent a disease or worsening of disease for this patient.
- ☐ This information will help to avoid unnecessary or inappropriate treatment, diagnostic procedures, preventive interventions or a referral for this patient.
